# Aquaporin-4 Autoantibodies in Neuromyelitis Optica: AQP4 Isoform-Dependent Sensitivity and Specificity

**DOI:** 10.1371/journal.pone.0079185

**Published:** 2013-11-15

**Authors:** Francesco Pisani, Angelo Sparaneo, Carla Tortorella, Maddalena Ruggieri, Maria Trojano, Maria Grazia Mola, Grazia Paola Nicchia, Antonio Frigeri, Maria Svelto

**Affiliations:** 1 Department of Bioscience, Biotechnologies and Biopharmaceutic and Center of Excellence in Comparative Genomics, University of Bari “Aldo Moro”, Bari, Italy; 2 Department of Neurosciences and Sense Organs, University of Bari Aldo Moro, Bari, Italy; 3 Dominick P. Purpura Department of Neuroscience, Bronx, New York, United States of America; Innsbruck Medical University, Austria

## Abstract

Neuromyelitis Optica (NMO) is an autoimmune demyelinating disease, characterized by the presence of autoantibody (NMO-IgG) to Aquaporin-4 (AQP4). NMO-IgG identification supports NMO diagnosis and several diagnostic tests have been developed, but their sensitivity is too variable, and some assay show low sensitivity. This impairs correct diagnosis of NMO. By cell based assay (CBA) we here evaluate the efficacy of different strategies to express AQP4 in mammalian cells in terms of: a) AQP4 translation initiation signals; b) AQP4 isoforms (M1 and M23) and fluorescent tag position; c) NMO serum concentration and AQP4 degradation. Our results demonstrate that when using AQP4-M1, the nucleotide in position −3 of the AUG greatly affects the AQP4-M1/M23 protein ratio, NMO-IgG binding, and consequently test sensitivity. Test sensitivity was highest with M23 expressing cells (97.5%) and only 27.5% with AQP4-M1. The fluorescent tag added to the N-terminus of AQP4-M23 considerably affected the NMO-IgG binding, and test sensitivity, due to disruption of AQP4 suprastructures. Furthermore, sera used at high concentration resulted in AQP4 degradation which affected test sensitivity. To further evaluate the reliability of the M23 based CBA test, samples of one NMO patient collected during about 2 years clinical follow-up were tested. The results of serum titer correlated with disease activity and treatment response. In conclusion, we provide a molecular explanation for the contrasting CBA test data reported and suggest the use of M23 with a C-terminus fluorescent tag as the proper test for NMO diagnosis.

## Introduction

Neuromyelitis Optica (NMO) is an autoimmune demyelinating disease characterized by the presence of autoantibody IgG1 type (NMO-IgG) in the patient serum [1]. The molecular target of NMO-IgG is Aquaporin-4 (AQP4), a plasma membrane water channel, particularly abundant in the perivascular astrocyte endfoot of the Central Nervous System (CNS) [2]. Moreover, NMO-IgG binding to AQP4, induces astrocyte injury and Blood Brain Barrier (BBB) breakdown causing NMO clinical symptoms [3], [4].

AQP4 is expressed as two major isoforms, called AQP4-M1 and AQP4-M23, forming heterotetramers in the plasma membrane. AQP4 tetramers further aggregate into supra-molecular structures named Orthogonal Arrays of Particles (OAPs) [5] whose size is correlated to the AQP4-M1/M23 protein ratio [6], [7].

Our group first reported that NMO-IgGs are not able to bind to a linear AQP4 epitope but specifically recognize conformational epitopes of AQP4-OAPs [8], [9]. These epitopes are generated by extracellular loop interaction, during heterotetramer aggregation required for OAP formations [9].

One of the three supportive criteria for NMO diagnosis is the identification of NMO-IgG in the patient serum [10]. Therefore, a test able to detect NMO-IgG, with high sensitivity and specificity is crucial in clinical practice for NMO diagnosis and treatment.

Several diagnostic tests have been developed based on different techniques. ELISA, radio-immunoprecipitation assay (RIPA), immunoprecipitation assay (IP), immunofluorescence (IF) on monkey cerebellum sections, and cell based immunofluorescence assays (CBA) on transfected cells have been proposed as tests for anti-AQP4 auto-autoantibody detection in patient’s serum [11], but their sensitivity is highly variable [12]. For instance, the sensitivity ranges from 35% to 91% for CBA test [12], [13], [14]. The reason for these differences remains completely unknown. Thus the detection of NMO-IgG is an issue still to be optimized considering that incorrect results of the test can impair correct diagnosis of NMO, especially in the earliest stages of the disease, and delay prompt initiation of disease-appropriate therapies. For some sera it is necessary to repeat the test, sometimes with a parallel use of the different detection systems described above, which is very expensive and time consuming.

In this paper we 1) provide a molecular explanation for the contrasting data reported on the CBA tests today available for NMO-IgG detection and 2) show the molecular strategy necessary to optimize NMO-IgG binding to AQP4 and obtain the highest sensitivity and specificity test (HSS-CBA test). In particular, we demonstrate that the best results are obtained with a CBA test based on the use of cells stably transfected with large fluorescent AQP4-OAPs. This paper represents a new molecular guideline, to set-up a high performance test for NMO-IgG detection.

## Materials and Methods

### Cloning of Human AQP4 in Mammalian Expression Vectors

Cloning of human AQP4-M1 and human AQP4-M23 wt CDSs in pTarget Expression Vector. Human AQP4-M1 and human AQP4-M23 CDSs were PCR amplified with the following primers, hAQP4-M1 forward: ggcatgagtgacagacccac (NCBI ID NM_001650.4); hAQP4-M23 forward: atcatggtggctttcaaagg (NCBI ID NM_004028.3); hAQP4 reverse: tcatactgaagacaatacct. AQP4-M1 and AQP4-M23 CDSs were then cloned starting from the −3 nucleotide from the ATG, to reconstitute the naturally occurring Translation Initiation Signal (TIS). PCR products were cloned into pTarget TA-Vector (Promega) and analyzed by DNA sequence analysis.

#### Cloning of hAQP4-M23 in pmCherry-N1

Human AQP4-M23 CDS was PCR amplified and cloned into pmCherry-N1 (Clontech) by BgLII and KpnI restriction sites to obtain human AQP4-M23 with C-terminal mCherry tag (AQP4-M23mCherry). The following primers were used, hAQP4-M23-BgLII forward: aaaaagatctatcatggtggctttcaaagg, and hAQP4-KpnI reverse: tttttggtaccgttactgaagacaatacct. hAQP4-M23 was cloned starting from the −3 nucleotide from the ATG, to reconstitute the naturally occurring Translation Initiation Signal (TIS).

#### Cloning of human AQP4-M23 in pcDNA3.1 NT-GFP

Human AQP4-M23 CDS was PCR amplified with the following primers, hAQP4-M23 forward: atcatggtggctttcaaagg, and hAQP4 reverse: tcatactgaagacaatacct and cloned into pcDNA3.1 NT-GFP Topo Vector (Invitrogen) according to the manual instructions, in order to obtain AQP4-M23 isoform with an N-terminal GFP tag (GFP-M23).

### Site Direct Mutagenesis of Human AQP4-M1

The nucleotide in position −3, (G^−3^) naturally present in wt human AQP4-M1 TIS, (G^−3^gcATGA^+4^, NCBI ID NM_001650.4) was changed into T, A and C, using Quikchange II Site-Direct Mutagenesis Kit (Stratagene) according to manual instructions. pTarget-human AQP4-M1, described in the previous paragraph, was used as template for the reaction. The following primers were used:

hM1G^−3^T for: gcCTCGAGACGCGTGATT**T**GCATGAGTGACAGACCCA

hM1G^−3^T rev: TGGGTCTGTCACTCATGCAAATCACGCGTCTCGAGgc

hM1G^−3^A for: gcCTCGAGACGCGTGATT**A**GCATGAGTGACAGACCCA

hM1G^−3^A rev: TGGGTCTGTCACTCATGCTAATCACGCGTCTCGAGgc

hM1G^−3^C for gcCTCGAGACGCGTGATT**C**GCATGAGTGACAGACCCAc

hM1G^−3^C Rev TGGGTCTGTCACTCATGCGAATCACGCGTCTCGAGgc

### Cell Cultures and Transfections

HeLa and V79 cell lines were grown in Dulbecco’s high glucose medium added with 10% fetal bovine serum and penicillin-streptomycin (Invitrogen). In order to perform transfection experiments the cells were plated at 90–95% confluence by Lipofectamine Reagent (Invitrogen). To obtain M23mCherry stable transfected cell line, cells were transfected with pmCherry-N1hAQP4-M23 and stable clones selected by G418 treatment. After selection, cells were further purified and enriched using MoFlo cell sorter of the Albert Einstein College of Medicine Flow Cytometry Core Facility.

### Antibodies

Anti-AQP4 antibodies were from Santa Cruz (goat polyclonal IgG), diluted 1∶500 for immunofluorescence and immunoblotting analysis. For some experiments, a rabbit anti-AQP4 serum, previously described [15], was used and diluted 1∶50 for immunofluorescence analysis. The secondary antibody utilized for immunoblotting analysis was a donkey anti-goat IgG peroxidase-conjugated from Santa Cruz at a dilution of 1∶5,000 whereas for immunofluorescence analysis it was a donkey anti-goat, donkey anti-rabbit or goat anti-human Alexa 488-conjugate at a dilution of 1∶1000.

### Patient Sera

In total 112 serum samples were tested for the presence of anti-AQP4 antibodies, forty from NMO patients and seventy two from controls. Controls included 56 patients with definite Multiple Sclerosis (MS), 10 with non-extensive inflammatory/infective myelitis, 1 with a recurrent form of myelitis, 1 with polyneuropathy, 1 with myopathy, and 3 healthy donors.

The forty NMO patients were selected as consecutively diagnosed patients according to Wingercuck’s 2006 criteria [10]. Alternative diagnoses were excluded by careful clinical and preclinical algorithm and eventually by an adequate period of observation. NMO patients were all relapsing. Two of these NMO patients had been previously tested and selected as AQP4-ab positives.

The diagnosis of MS was established according to the McDonald criteria [16]. 15 samples from one NMO patient, collected during about 2 years of clinical follow-up, were tested individually. This patient was treated with Cascade Plasma Filtration therapy (CPFT), and Mitoxantrone. Sera were used at dilutions of 1∶50–1∶8000 for immunofluorescence analysis and slides were examined independently by two investigators who were not aware of the clinical or laboratory information of studied patients.

### AQP4 Immunofluorescence Analysis

Transfected HeLa or V79 cells were first fixed with 4% formaldehyde in PBS (Sigma, HT5014) for 10 minutes, washed 3 times with PBS, permeabilized with 0.3% TritonX-100 in PBS for 10 minutes and finally saturated with PBS-0.1% gelatin for 10 minutes. Cells were then incubated with commercial anti-AQP4 antibody (Santa Cruz Biotechnology, AQP4 (C-19): sc-9888) for 30 minutes, washed 3 times with PBS and incubated for 30 minutes with Alexa-conjugated secondary antibodies, washed and finally mounted in PBS-glycerol (1∶1) pH 8.0, containing 1% n-propyl gallate. Immunostained cells were screened with a photomicroscope equipped for epifluorescence (DMRXA; Leica) and digital images were obtained with a DMX 1200 camera (Nikon, Tokyo, Japan). Quantitative AQP4 expression was obtained using ImageJ software and using gray scale converted images.

### Total Internal Reflection Fluorescence (TIRF) Microscopy Analysis to Measure AQP4 Cell Surface Expression Levels in Transfected Cells

AQP4 transfected cells were stained with commercial AQP4 antibodies as described above and analyzed for cell surface expression as follow. A Nikon laser TIRF setup was used, consisting of a 488 nm argon laser mounted on a Nikon laser TE2000U microscope, which also allows phase-contrast and epifluorescence techniques to be combined with TIRF technology. An incidence angle greater than the critical angle was achieved by the use of a 100X CFI Plan Apo of 1.45 numerical aperture. Fluorescence excited by TIR evanescent field (∼100 nm) was collected with the same objective, and images were collected by a cooled charge-coupled device camera (Hamamatsu Orca). The TIRF signal, corresponding to AQP4 expressed at the cell surface, was measured in ten independent areas of transfected cells. Images were first converted to gray scale and gray values were measured by ImageJ software.

### Ethics Statements

#### Animals

All experiments conformed to international guidelines on the ethical use of animals were designed to minimize the number of animals used and their suffering (European Council Directive of 24 November 1986 (86/609/EEC)). Experiments in this study were approved by the Italian Health Department (Art. 9 del Decreto Legislativo 116/92).

The mice used here were bred in the approved facility at the University of Bari. Mice were kept under a 12 hours dark to light cycle, constant room temperature and humidity (22±2°C, 75%), with food and water ad libitum, and supplied with environmental enrichment materials, such as toys and shelters.

#### Human subjects

All subjects gave their written informed consent to the study, which was approved by the institutional review board of the University of Bari.

### Immunofluorescence on Mice Brain Sections

Adult CD1, wild type and AQP4 null mice [17] were anesthetized by intraperitoneal injection of urethane (1.2 g/kg body weight) and the brain dissected and rapidly frozen in liquid nitrogen in OCT medium and sectioned (8 µm). Brain sections were stained with patient sera 1∶1000 and anti-AQP4 rabbit serum 1∶50, in PBS containing 0.1% gelatin. Anti-human Alexa 488-conjugate and Anti-rabbit Alexa 594-conjugate, at a dilution of 1∶1000, were used as secondary antibodies.

### Cell based Assay and NMO-IgG Test

Cell based assay for NMO-IgG binding was performed using live Hela or V79 cells, in PBSCa^2+^Mg^2+^0.1% gelatin, using 1∶10 to 1∶8000 NMO serum, at RT for 1 h. After incubation with NMO serum, cells were washed and secondary antibodies (anti-human 488 Alexa-Fluor) 1∶1000, was added. Cells were washed, fixed for 10 minutes with 4% formaldehyde in PBS (Sigma, HT5014) and finally mounted in PBS-glycerol (1∶1) pH 8.0, containing 1% n-propyl gallate. NMO-IgG detection by Euroimmune test (Cat number, FA 1111-1003) was performed according to the instructions. To evaluate the effect of cell fixation on NMO-IgG binding 12 NMO sera representative of previously identified major conformational epitopes [9] were tested. Cells were first fixed for 1 minute with 4% formaldehyde in PBS (Sigma, HT5014) and then processed for IF.

### SDS-PAGE, and Western Blotting

Tris-Glycine SDS-PAGE gel and the immunoblotting were performed as described [8].

### Statistical Analysis

Statistical analysis (means, medians, range, standard deviations), significance of group differences were evaluated using GraphPad Prism 5 (GraphPad, San Diego, USA) by student’s t-test. A p value <0.05 was considered statistically significant. Sensitivity and Specificity 95% confidence intervals were computed using exact methods. The likelihood ratio was calculated as reported [18].

## Results

### CBA Sensitivity is Affected by the use of different TIS for the AQP4-M1 Coding Sequence

**Figure 1:**
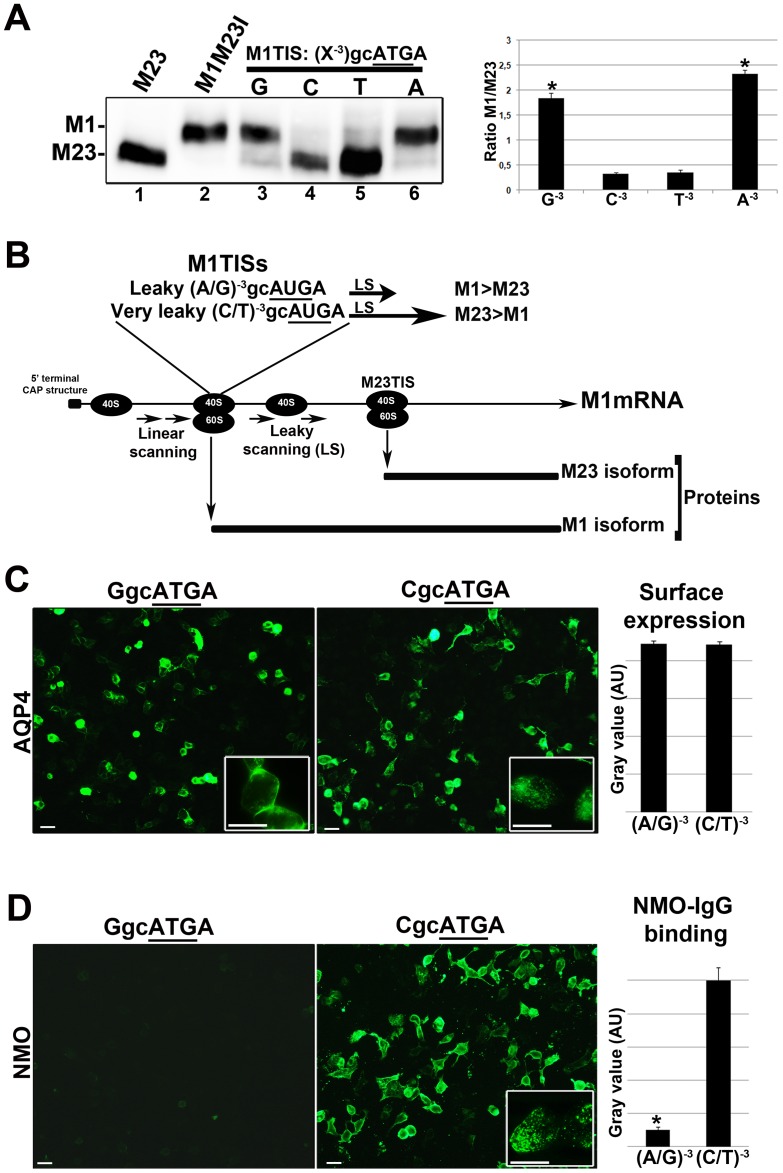
Correlation between AQP4-M1 CDS cloning strategy, LS frequency, the AQP4-M1/M23 ratio and NMO-IgG binding. (A): Immunoblot experiment performed using commercial AQP4 antibodies on cells transfected with different AQP4 expressing constructs. From the left: lane 1, AQP4-M23 exclusively producing AQP4-M23, lane 2, AQP4-M1M23I (with the methionine in position 23 mutated into Isoleucin), exclusively producing AQP4-M1, lane 3–6: the four constructs under analysis with G or C or T or A, in the N^−3^position, as indicated. On the right: densitometric analysis of the AQP4-M1 and AQP4-M23 bands reported as the AQP4-M1/M23 ratio (n = 4), *P<0.01, (A^−3^/G^−3^) vs (C^−3^/T^−3^). (B): Illustration showing the cap-dependent translation and relative LS frequency on AQP4-M1 mRNA depending on TIS. (C) Left: immunofluorescence experiment, using AQP4 commercial antibody, performed on AQP4-M1 with TIS (G)^−3^gcAUGA^+4^, and AQP4-M1 with TIS (C)^−3^gcAUGA^+4^, transfected cells. Right: quantitative analysis of the fluorescence signal obtained by TIRF microscopy of transfected cells with vectors having a purine (A^−3^/G^−3^) or a pyrimidine (C^−3^/T^−3^) at the N^−3^, (n = 4). (D) Left: immunofluorescence experiment performed on transiently transfected cells using NMO sera (collection of 20 NMO sera). The level of NMO-IgG binding is very low in cells transfected with AQP4-M1 with TIS (G)^−3^gcAUGA^+4^, while it is high in cells transfected with AQP4-M1 with TIS (C)^−3^gcAUGA^+4^. Right: quantitative analysis of NMO-IgG binding on cells transfected with vectors having a purine (A^−3^/G^−3^) or a pyrimidine (C^−3^/T^−3^) at the N^−3^, analyzed by immunofluorescence (n = 4). *p<0.01. Magnification bar 10 µm.

**Figure 2:**
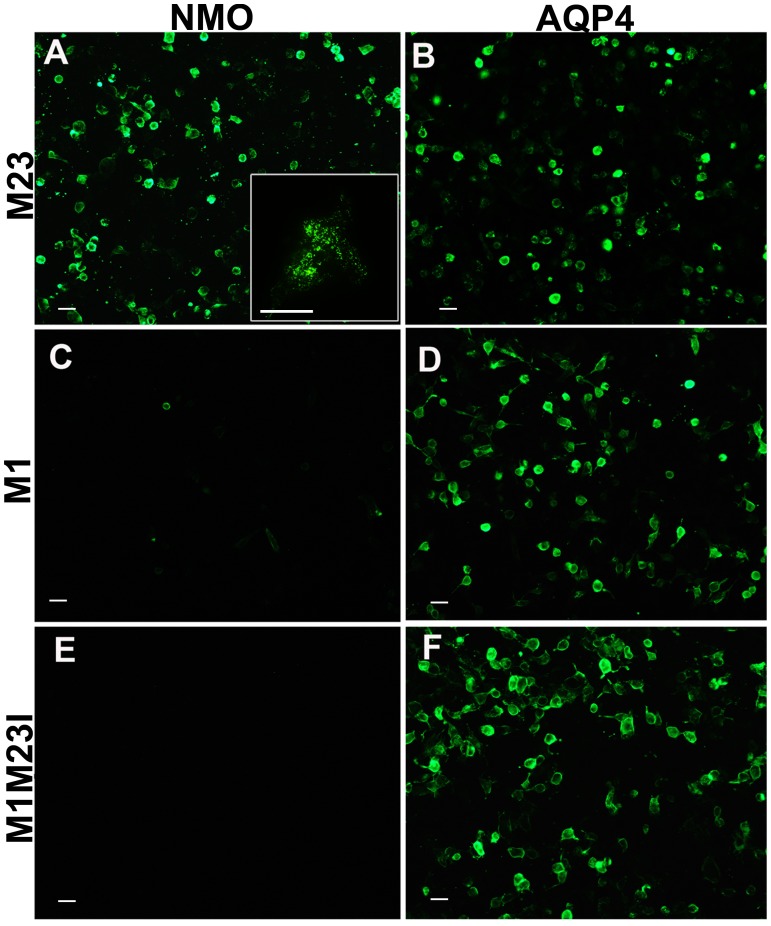
Changes in sensitivity of the CBE test using cells transfected with different AQP4 isoforms. Immunofluorescence analysis performed using NMO sera (NMO) and AQP4 commercial antibodies (AQP4) on cells transfected with AQP4-M23 (M23), WT AQP4-M1 (M1) and the mutated form of AQP4-M1 (M1M23I). The binding is very efficient for cells transfected with AQP4-M23, strongly recognized by NMO sera (collection of 20 NMO sera), whereas it is weak for cells transfected with AQP4-M1. No or very weak staining was detectable for cells transfected with M1M23I. Magnification bar, 10 µm.

Most of the CBA tests reported for NMO-IgG detection are based on the use of the AQP4-M1 coding sequence (CDS) [19], [20], [21], [22], which is also able to produce the AQP4-M23 isoform through a mechanism of leaky scanning (LS) [6], [23]. LS frequency is regulated by the nucleotide N^−3^ [24], [25]. N^−3^ is a G (G^−3^gcAUGA^+4^) in WT AQP4-M1 TIS. Experiments have been here performed to demonstrate that changing the G^−3^ nucleotide in AQP4-M1 TIS affects the LS frequency and AQP4-M1/M23 ratio and OAP size as a consequence. This in turn affects NMO-IgG binding and therefore the sensitivity of the CBA test. Single site mutations at the G^−3^ nucleotide were then performed, and the AQP4-M1/M23 ratio determined by immunoblot (Fig. 1A). WT AQP4-M1 TIS (with a G^−3^) produced low levels of AQP4-M23 protein confirming that the LS mechanism is occurring but with a low frequency [6], [23]. Substituting G^−3^ with a pyrimidine (C or T) increased dramatically the LS frequency allowing the highest AQP4-M23 synthesis, with the virtual absence of AQP4-M1 protein. Substituting G^−3^ with the purine A did not produce changes in the AQP4-M1/M23 ratio compared to WT. These results are in agreement with the literature data reporting that a TIS with a (C/T) ^−3^ is very inefficient for translation initiation, or “leaky” [25], during the cap-dependent translation mechanism (Fig. 1B). We then tested whether LS efficiency is able to affect NMO-IgG binding to AQP4-M1 transfected cells. To this end, immunofluorescence experiments were first performed on cells transfected with AQP4-M1 TIS having a pyrimidine (C^−3^ or T^−3^) or a purine (A^−3^ or G^−3^), using commercial AQP4 antibody and NMO sera, to measure, surface expression (by TIRF microscopy) and NMO-IgG binding, respectively. Results showed that the surface expression is not affected by changing AQP4-M1 TIS (Fig. 1C) while efficiency of NMO-IgG binding is tightly correlated to the nucleotide N^−3^, as expected (Fig. 1D). Using NMO sera, a low level of fluorescent signal occurred in AQP4-M1 transfected cells having a purine A/G^−3^ while the specific signal strongly increased using AQP4-M1 transfected cells having a pyrimidine C/T^−3^. Given that, in many cases the nucleotide N^−3^ is randomly defined by the vector sequence, or defined by the forward PCR primer sequence, these results indicate that the possibility to have a purine or a pyrimidine at N^−3^ seriously affects the sensitivity of CBA. Moreover, these data further confirm that NMO-IgG is specific for AQP4-OAP structure and not for AQP4 tetramer [8]. The CBA sensitivity has since been quantitatively analyzed based on the AQP4-M1/M23 expression ratio in the cells used for the assay (Table 1). We tested 112 sera, which included 40 NMO sera, on cells transfected with: 1) AQP4-M23 expressing vector producing only the OAP forming AQP4-M23 isoform, 2) AQP4-M1 expressing vector, having a WT TIS (G^−3^) and therefore producing AQP4-M1>AQP4-M23 and 3) a AQP4-M1 mutant (M1M23I) exclusively producing AQP4-M1 since AQP4-M23 synthesis via LS is prevented (Fig. 1A) [23]. The results showed that test sensitivity was 97.5% in the AQP4-M23 based test, 27.5% in the AQP4-M1 based and 0% in the AQP4-M1M23I based one, indicating the AQP4-M23 expressing vector as the gold standard for a high sensitivity test. Figure 2 shows a representative immunofluorescence experiment, relative to data reported in table 1. To confirm that AQP4-M23 and AQP4-M1M23I display similar cell surface expression, we performed TIRF microscopy (figure S1). Therefore, the absence of NMO-IgG binding on M1M23I expressing cells was not due to a sub-cellular localization of this mutant.

**Table 1:** Summary of the results obtained in terms of sensitivity and specificity for testing sera by immunofluorescence on cells transfected with AQP4-M23 or AQP4-M1 (AQP4-M1>M23) or AQP4-M1 (M1M23I).

Patient sera(n = 112)	AQP4-M23 based test(M23 isoform)	AQP4-M1 based test(M1>M23)	AQP4-M1M23I based test(M1 isoform)
NMO (n = 40)	Positive: 39	Positive: 11	Positive: 0
**Sensitivity**	39/40 = **97.5%** (95% CI: 87%–100%)	11/40 = **27.5%** (95% CI: 15%–44%)	0/40 = **0%** (95% CI: 0%–9%)
Other diagnosis andhealthy donors (n = 72)	Positive: 0	Positive: 0	Positive: 0
**Specificity**	72/72 = **100%** (95% CI: 95%–100%)	72/72 = **100%** (95% CI: 95%–100%)	72/72 = **100%** (95% CI: 95%–100%)
**Likelihood ratios**	LR+: ∞; LR−: 0.025	LR+: ∞; LR−: 0.725	LR+: ∞; LR−: 1

Note that the sensitivity is 97.5% and specificity is 100%.

### Sensitivity is Affected by the Presence of a Fluorescent Tag at the N-terminus of AQP4

**Figure 3:**
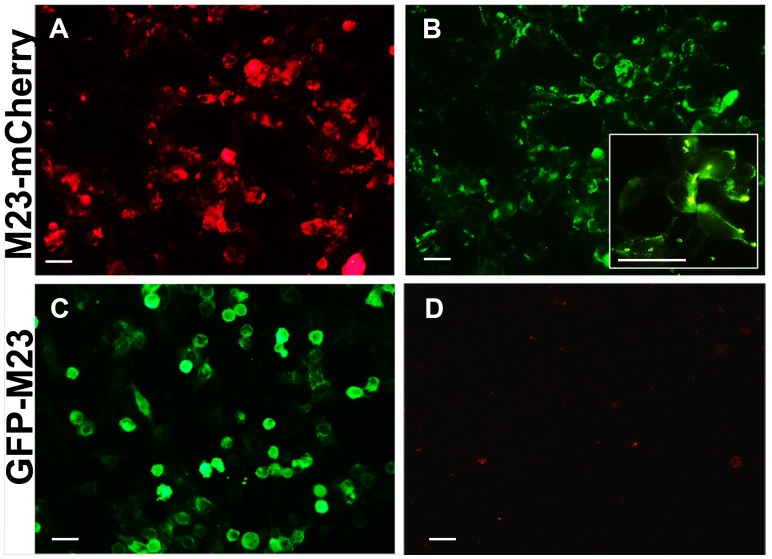
Impact of a fluorescent AQP4 tag on NMO-IgG detection. Fluorescence of mCherry tagged at the AQP4 C-terminus (A) and GFP tagged at the AQP4 N-terminus (C). Immunofluorescence experiments conducted with NMO sera (collection of 20 NMO sera) on AQP4-M23mCherry (B) and GFPAQP4-M23 (D). The plasma membrane NMO-IgG staining is highlighted in the inset in B. Magnification bar, 10 µm.

Several studies report the use of cells expressing AQP4 as fusion protein with a fluorescent tag, mainly GFP, to detect NMO-IgG in patient sera [26], [27]. Adding a tag to the N-terminus of AQP4 can affect OAP formation [9] therefore we tested the NMO-IgG binding to AQP4-M23 with an N-terminal GFP tag or with a C-terminal mCherry tag (Fig. 3). The results obtained using all 40 NMO sera showed that no NMO-IgG staining was observed when AQP4 is tagged at the N-terminus whereas NMO-IgG binding is completely unaffected by the presence of a C-terminus tag. These data, in agreement with those previously reported using a lower number of sera [9], showed that the presence of an N-terminus tag on the AQP4 sequence is not compatible with the detection of NMO-IgG whereas the presence of a C-terminal tag did not affect the sensitivity of the test (Table 2).

**Table 2:** NMO-IgG binding on AQP4-M23 with C- or N- terminal fluorescent tag.

Patient sera (n = 112)	Positive on M23-mCherry	Positive on GFP-M23
NMO (n = 40)	Positive: 39	Positive: 0
**Sensitivity**	39/40 = **97.5%** (95% CI 87%–100%)	0/0 = **0%** (95% CI 0%–9%)
Other diagnosis andhealthy donors (n = 72)	Positive = 0	Positive = 0
**Specificity**	72/72 = **100%** (95% CI 95%–100%)	72/72 = **100%** (95% CI 95% 100%)
**Likelihood ratios**	LR+: ∞; LR−: 0.025	LR+: ∞; LR−: 1

### Sensitivity is Affected by High NMO Serum Concentration via AQP4 Degradation

**Figure 4:**
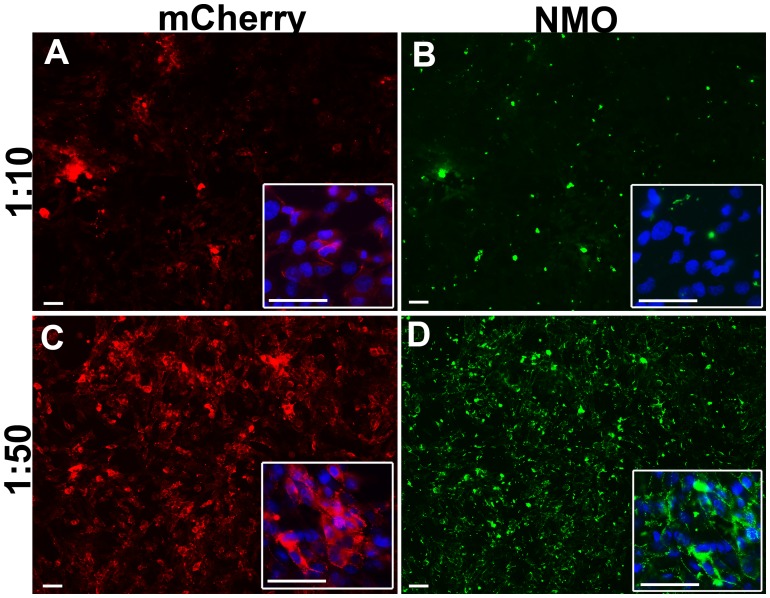
High NMO serum concentration induces false NMO-IgG negative via AQP4 degradation. Immunofluorecence experiment using NMO serum (NMO) on AQP4-M23mCherry expressing cells (mCherry). High NMO serum concentration (1∶10 dilution) incubated for 1h at RT, caused AQP4 signal reduction (A) and very little NMO staining (B), while cells incubated with the same serum but at a lower concentration (1∶50 dilution) were strongly AQP4 positive (C) and heavily NMO stained (D). Nuclei stained with DAPI in blue. Magnification bar 10 µm.

The CBA test was performed on unfixed cells [28] since we have demonstrated that classical fixation (10 minutes with 4% formaldehyde in PBS) affects the conformational epitope [8], [9]. However, degradation of AQP4, induced by NMO-IgG, may occur in living transfected cells [3]. We have hypothesized that this mechanism may also occur during CBA, therefore affecting test sensitivity. We have verified this hypothesis using NMO sera at different concentrations (Fig. 4).

Incubating AQP4-M23mCherry expressing cells with a high concentration (1∶10 dilution) of a strongly NMO-IgG positive serum, resulted in a strong decrease in AQP4 signal and mainly no NMO-IgG staining, while diluting the same serum at 1∶50, cells maintained high levels of AQP4 and were strongly stained by NMO-IgG. This data suggests that high NMO serum concentration induces AQP4 degradation at RT in PBS, consequently test sensitivity decreases and therefore a false negative may occur.

### AQP4-M23mCherry Cell based Test Correlates with Disease Activity and Response to Treatment in NMO Patients as well as showing Very High Technical Sensitivity

All the results shown in the previous paragraphs strongly indicated a CBA test, based on the use of the large OAP forming isoform AQP4-M23 having a C-terminal fluorescent tag, as the highest sensitivity and specificity test (HSS-CBA test). To further evaluate the reliability of our HSS-CBA test, 15 samples from one NMO patient, collected over approximately 2 years of clinical follow-up, were individually tested by limiting dilution, using the HSS-CBE test and a commercial kit based on monkey cerebellum sections, in parallel. Table 3 reports the results of this follow-up analysis with the two methods. For low titer samples the commercial kit was often negative, while our HSS-CBA test was always able to detect a positive signal for all the samples, confirming its much higher sensitivity. The serum titer results correlated with disease activity and treatment response. The disease exacerbation phase is characterized by the highest titers, whereas Cascade Plasma Filtration therapy (CPFT) causes a progressive titer reduction paralleling clinical improvement, as does Mitoxantrone partially.

**Table 3:** Follow-up of NMO patient sera analyzed using a commercially available NMO-IgG kit and the HSS-CBA test.

Serum	Disease activity*	Ongoing treatment	CommercialNMO-IgG test	AQP4-M23mCherryTest	NMO serumtiter
1	Yes (severe myelitis)	None	Positive	Positive	1∶8000
2	Yes (myelitis)	None	Negative	Positive	1∶2000
3	Yes (myelitis)	Mitoxantrone	Positive	Positive	1∶1500
4	Yes (myelitis)	Mitoxantrone	Negative	Positive	1∶1000
5	Yes (myelitis)	Mitoxantrone	Negative	Positive	1∶1000
6	Yes (optic neuritis & myelitis)	Mitoxantrone	Negative	Positive	1∶2000
7**	Yes (severe myelitis)	None (Pre CPFT)	Positive	Positive	1∶2000
8	Yes	CPFT (1st session)	Positive	Positive	1∶1000
9	Yes	CPFT (2nd session pretreatment sample)	Positive	Positive	1∶1000
10	Yes	CPFT (2nd session)	Negative	Positive	1∶500
11	Yes	CPFT (3rd session pretreatment sample)	Negative	Positive	1∶200
12	Yes (mild improvement)	CPFT (3rd session)	Negative	Positive	1∶100
13	Yes (improvement)	CPFT (6th session pretreatment sample)	Negative	Positive	1∶200
14	Yes (improvement)	CPFT (6th session)	Negative	Positive	1∶100
15	Yes (optic neuritis and myelitis)	None	Positive	Positive	1∶2000

Note the difference between NMO-IgG evaluated using the test on monkey cerebellum or the HSS-CBA test. 9/15 samples were negative with the cerebellum-based test, while all the samples were positive on cells. Note the good correlation between the cell based test and Cascade Plasma Filtration therapy (CPFT) treatment. Serum titer was measured by limiting dilution.

* Yes: exacerbation phase of the disease; No: inactivity phase of the disease.

** Samples 7–14 were collected during a 15 day cycle of CPFT (one therapeutic session every other day) planned because of a severe exacerbation. Serum samples were collected before and after each CPFT session.

### False NMO-IgG Positive may Occur using a Tisssue-based IF Test

**Figure 5:**
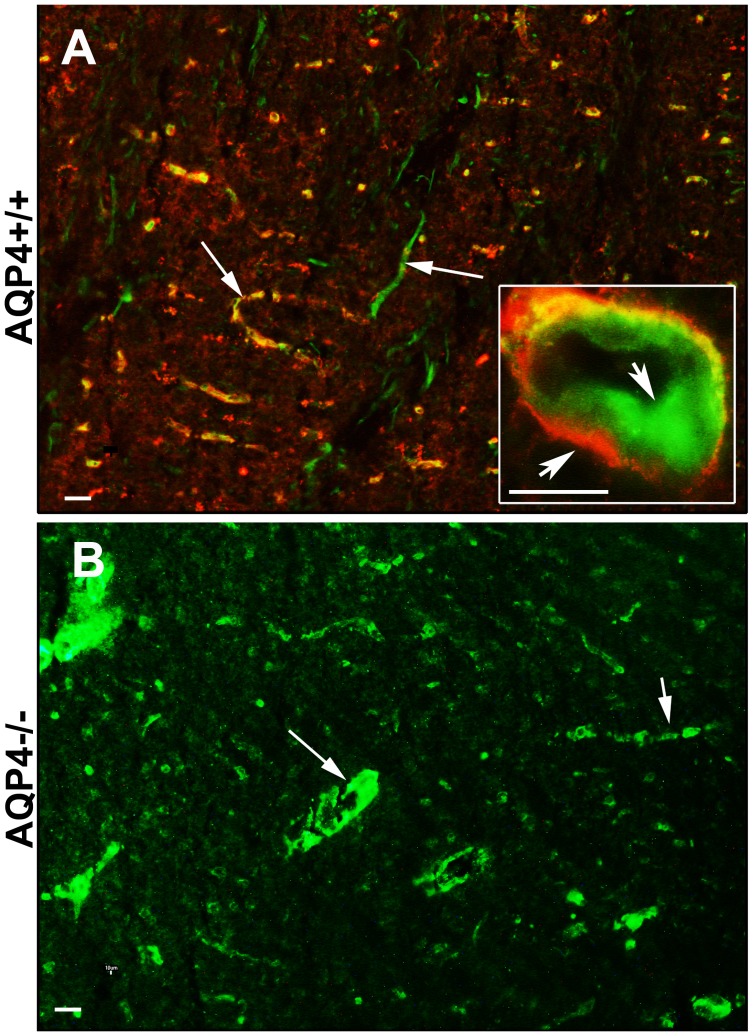
AQP4 independent staining of false NMO-IgG positive sera. WT (A) and AQP4 knockout (B) brain sections were stained with a serum showing NMO IgG-like staining at low magnification. Inset (A) shows that the serum staining (green) is restricted to endothelial cells of the brain capillaries and not to astrocyte processes stained red by AQP4 antibodies. (B) Staining of AQP4−/− mouse endothelial cells of the serum under analysis. Magnification bar 10 µm.

In order to evaluate possible limitation in the use of a tissue based test, 65 sera, tested by CBA, were then tested using the commercial tissue-based IF test (TB-A) (Euroimmune monkey cerebellum). This test allows the identification of NMO-IgG as perivascular staining at astrocyte end feet. As reported in table 4, this test shows low sensitivity and specificity. The specificity was particularly low (41%), indicating that the positivity of this test is not strongly associated with NMO diagnosis. To understand factors that could so seriously affect a TB-A, we tested some sera that were positive in a TB-A and negative in HSS-CBA, on AQP4 null mice brain. Surprisingly, co-immunofluorescence experiments performed using those sera and AQP4 antibodies on AQP4+/+ and AQP4−/− mouse brain (Fig. 5 A) clearly demonstrated the presence of endothelial, NMO IgG-like but AQP4-independent, staining of some NMO sera demonstrating that TB-A, may have serious limitations especially due to false positive results. It should be noted that a pre-absorption step may reduce the non specific binding of antibodies but this procedure may also affect the binding of low affinity AQP4 autoantibodies as well and reduce the sensitivity of the test.

**Table 4:** NMO-IgG detection using the monkey cerebellum based IF test.

Patient sera (n = 65)	Positive	Negative
NMO (n = 24)	12	12
	**Sensitivity** 12/24 = **50%** (95% CI: 29%–71%)	
Other diagnosis (n = 41)	24	17
	**Specificity** 17/41 = **41%** (95% CI: 26%–58%)	
**Likelihood ratios**	LR+: 0.84; LR−: 1.22	

Note the very low sensitivity and specificity of this test.

## Discussion

Since the discovery of AQP4 as the target for NMO-IgG many diagnostic tests have been developed. However, especially those based on transfected cells (CBA) have shown a wide range of sensitivity, from 35 to 91% [12], [13], [14], but no rational interpretation has been proposed for these differences. Therefore, to date, a negative result, cannot rule out the diagnosis of NMO, and sometimes repeated testing is needed thus delaying adequate disease-specific treatment [29]. Serological assay methodologies based on the use of AQP4-M1 isoform are those that largely vary in sensitivity [19], [20], [21], [22]. In the first reports, mainly human AQP4-M1 was used as suggested by the pioneer paper of Lennon [2] and only recently, when it was demonstrated that OAPs are the main target of NMO-IgG [8], has the use of AQP4-M23 become more widely used [21]. Furthermore, although there is evidence reporting binding of NMO-IgG to AQP4 monomer and tetramer in HEK293 cells [30], more recent data confirm the strong binding capacity of NMO-IgG to M23-OAPs [31], [32], [33], [34], [35].

Here we demonstrate that when using AQP4-M1 the nucleotide in position −3 of the AQP4-M1 A^+1^UG, greatly affects LS frequency, the AQP4-M1/M23 protein ratio, NMO-IgG binding, and consequently test sensitivity. Therefore, it is extremely important how the AQP4-M1 CDS is cloned. All the data in the present study support the use of AQP4-M23 as the gold standard for a high sensitivity NMO test allowing a much higher sensitivity (97.5%) with respect to AQP4-M1 (27.5%). Another important issue to be highlighted in view of preparing the AQP4 expressing vector for CBA is the position of the fluorescent tag. If it is placed at the C-terminal of the AQP4-M23 isoform it does not affect the sensitivity of the test, whereas if placed at the N-terminal it affects OAP formation and therefore test sensitivity. For example, McKeon and colleagues [36] reported a very low sensitivity (33%), in IP based tests, which is probably due to a GFP tag linked at the AQP4 N-terminal (GFP-AQP4).

In a few reports a high sensitivity has been obtained using AQP4-M1. For instance, Jarius and colleagues [19], developed a CBA test using “full length human AQP4”, likely corresponding to human AQP4-M1 isoform. They reported 78% sensitivity using NMO serum at 1∶10 dilution. This result appears to be in contrast with an OAP-specific NMO-IgG binding. However, going into the detail of the AQP4-M1 vector used in the Jarius paper, it is possible to conclude that AQP4-M1 CDS was cloned with a C in position −3 (C^−3^) that induced a strong LS mechanism therefore producing high levels of AQP4-M23 (see Fig. 1). However, Kalluri et al reported [20] a relatively high sensitivity (82%) for a flow cytometry assay using a G^−3^ of AQP4-M1 probably due to the normally higher sensitivity of the flow cytometry compared to the CBA.

A multicenter study has recently reported a comparison of the sensitivity of different anti AQP4 antibody assays [37]. This study further confirms that the variability in sensitivity is likely to depend on the AQP4 isoform used. The highest sensitivities reported were yielded by flow cytometry (77%) or by CBA (73%) using cells expressing both AQP4-M1 and AQP4-M23 isoforms [37].

Importantly, a very recent work demonstrates that re-testing NMO-patients the best results (sensitivity and specificity) were obtained using M23 expressing cells and FACS assay [38]. Finally, the commercial ELISA kit has been reported [22], [37], [39], [40], [41] to have generally a low sensitivity suggesting inadequate binding of NMO-IgG to purified AQP4.

We propose the use of a stable transfected cell line, expressing fluorescent AQP4-M23 (with a C-terminal fluorescent tag) as a gold standard cell line for a high performance NMO-IgG identification test with a high sensitivity/specificity (97.5% and 100%, respectively). This test also showed a high technical sensitivity, allowing the identification of very low NMO-IgG titer in patient serum, which is crucial for NMO diagnosis and monitoring. The use of a C-teminal fluorescent tag is recommended for two reasons. First, to evaluate whether the high serum concentration determines degradation of AQP4, as demonstrated in figure 4. Second, when using a stable cell line there is a spontaneous tendency to lose protein expression during cell passages. Thus, the fluorescent tag will allow rapid monitoring of the expression levels of AQP4 in the cell line and proper evaluation of the immunoflurescence results. As a final point, although there are some reports [36] regarding the presence of GFP antibodies in a few human samples (∼5%) this should not be a serious issue when using non permeabilized cells since the C-terminal epitope, and thus the fluorescent tag, of AQP4 is intracellular.

Serum concentration is a critical parameter that must be taken into account when a CBA test is used to avoid false negatives. This a serious issue when living cells are used that can be solved if, instead, fixed cells are used. However, to improve standardization of the AQP4 antibody testing, we found that fixing the cells for 1 minute with 4% formaldehyde in PBS does not significantly affect the antibody binding to each of the three groups of key immunodominant epitopes (Figure S2) previously identified [9].

Finally, tissue based IIF and especially the ELISA based test are strongly discouraged due to false NMO-IgG positives (due to low specificity) or negatives (due to low sensitivity).

In conclusion, this study gives important advice on several key aspects related to NMO IgG detection which is essential for early NMO diagnosis and treatment. Moreover, it demonstrates that the CBA test based on the use of the large OAP forming isoform AQP4-M23 with a C-terminal fluorescent tag is the proper test for NMO-IgG detection.

## Supporting Information

Figure S1 **TIRF microscopy quantitative analysis of AQP4-M1M23I and AQP4-M23 cell surface expression.** Left: representative immunofluorescence TIRF microscopy images of HeLa cells transiently transfected with AQP4-M1M23I and AQP4-M23 revealed by commercial AQP4 antibody. Note the punctuate staining of M23 expressing cells (i.e OAPs) compared to the diffuse staining of M1M23I expressing cells (no OAPs expression). Right: Quantification of the TIRF signal at the cell surface (n = 10). Magnification bar 2.5 µm.
(TIF)

Figure S2 **Mild fixation does not affect NMO-IgG binding to the major immunodominant AQP4 epitopes.** A: Representative immunofluorescence using NMO sera of three major conformational epitopes [9] (NMO1–3) on living or mildly fixed AQP4 (M23) expressing cells (Green: NMO-IgG staining; Blue: cell nuclei). Right, cartoon showing a schematic representation of the contribution of the extracellular loops in the generation of each immunodominat epitope of the OAP. B: Quantitative analysis of NMO-IgG to M23 expressing cells. Note that the number of cells stained by NMO-IgG, measured by green stained cells (%), is not affected by mild fixation (n = 3–5 different sera for each group).
(TIF)
