# Supplementary figures and images for: Aquaporin-4 Autoantibodies in Neuromyelitis Optica: AQP4 Isoform-Dependent Sensitivity and Specificity
Source: PLoS One. 2013 Nov 15;8(11):e79185. doi: 10.1371/journal.pone.0079185 (PMC3829826; doi:10.1371/journal.pone.0079185)

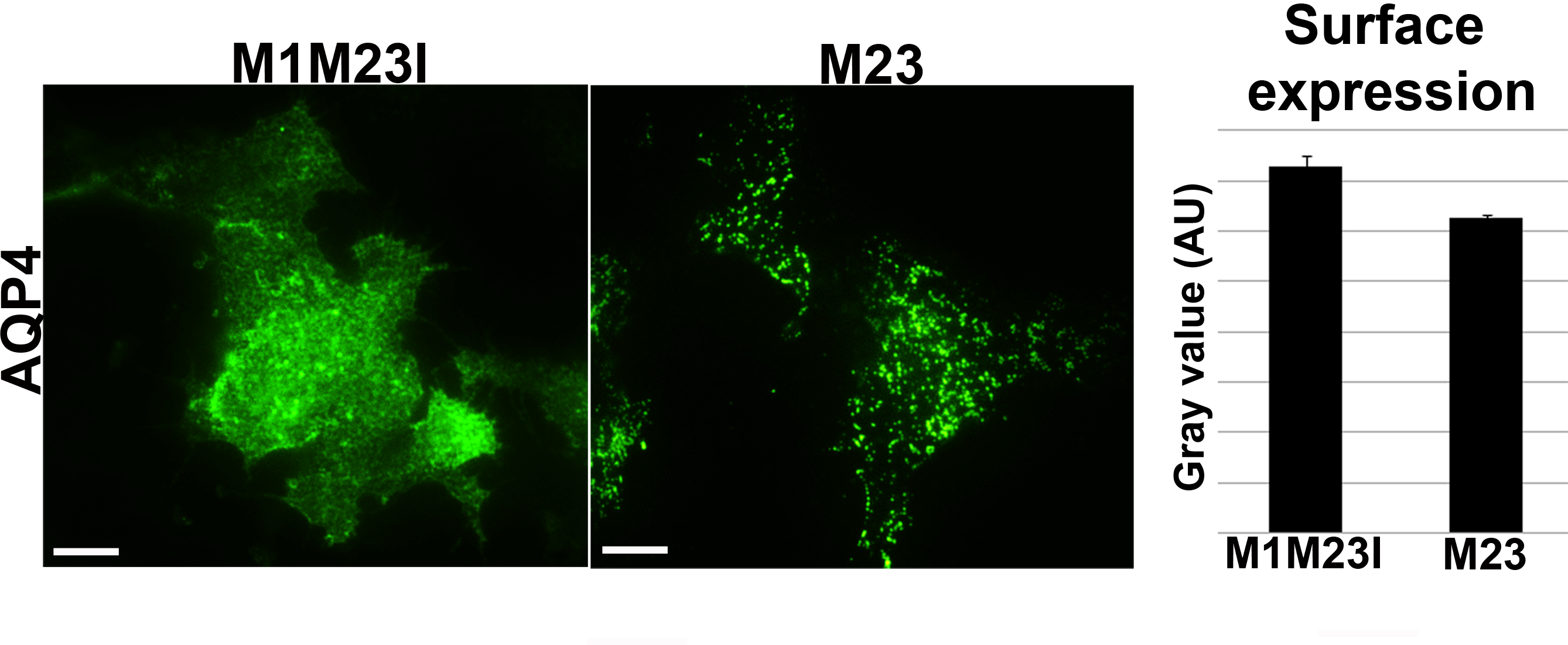

Supplement: Figure S1 — TIRF microscopy quantitative analysis of AQP4-M1M23I and AQP4-M23 cell surface expression. Left: representative immunofluorescence TIRF microscopy images of HeLa cells transiently transfected with AQP4-M1M23I and AQP4-M23 revealed by commercial AQP4 antibody. Note the punctuate staining of M23 expressing cells (i.e OAPs) compared to the diffuse staining of M1M23I expressing cells (no OAPs expression). Right: Quantification of the TIRF signal at the cell surface (n = 10). Magnification bar 2.5 µm. (TIF) [file pone.0079185.s001.tif]

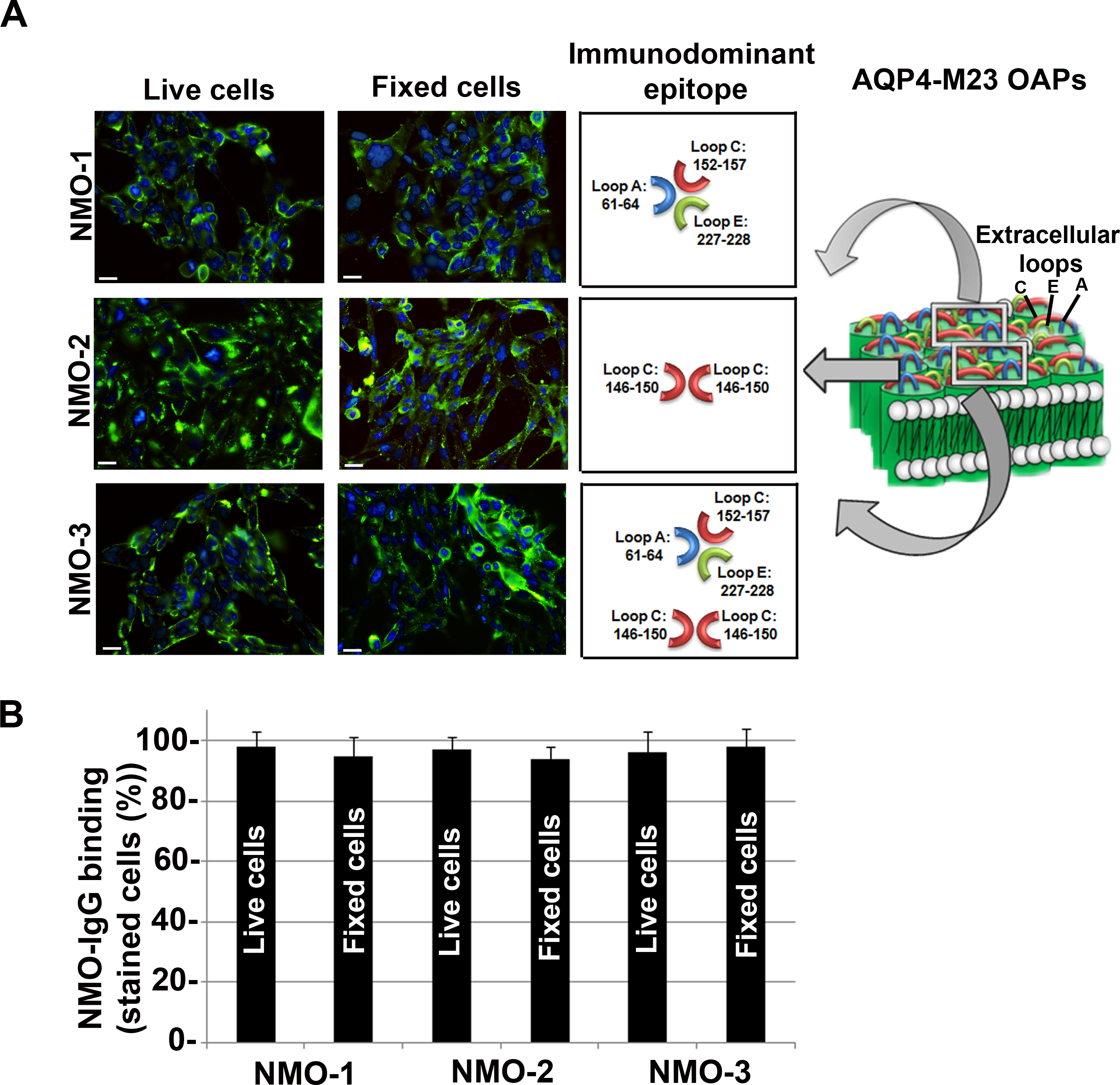

Supplement: Figure S2 — Mild fixation does not affect NMO-IgG binding to the major immunodominant AQP4 epitopes. A: Representative immunofluorescence using NMO sera of three major conformational epitopes [9] (NMO1–3) on living or mildly fixed AQP4 (M23) expressing cells (Green: NMO-IgG staining; Blue: cell nuclei). Right, cartoon showing a schematic representation of the contribution of the extracellular loops in the generation of each immunodominat epitope of the OAP. B: Quantitative analysis of NMO-IgG to M23 expressing cells. Note that the number of cells stained by NMO-IgG, measured by green stained cells (%), is not affected by mild fixation (n = 3–5 different sera for each group). (TIF) [file pone.0079185.s002.tif]
